# Reference biometry of foetal brain by prenatal MRI and the distribution of measurements in foetuses with ventricular septal defect

**DOI:** 10.1080/07853890.2021.1969590

**Published:** 2021-08-20

**Authors:** Feng Xia, Yu Guo, Hua He, Peiwen Chen, Jianbo Shao, Wei Xia

**Affiliations:** a Department of Radiology, Maternal and Child Health Hospital of Hubei Province, Wuhan, China; b Department of Imaging Center, Wuhan Children's Hospital (Wuhan Maternal and Child Healthcare Hospital), Tongji Medical College, Huazhong University of Science and Technology, Wuhan, China; c Department of Obstetrics, Maternal and Child Health Hospital of Hubei Province, Wuhan, China; d Department of Ultrasound, Maternal and Child Health Hospital of Hubei Province, Wuhan, China

**Keywords:** Foetal brain, magnetic resonance imaging, prenatal development, biometry, ventricular septal defect

## Abstract

**Objective:**

To provide the reference biometric measurements of the normal foetal brain by prenatal MRI and describe the distribution of measurements in the foetuses with ventricular septal defect (VSD).

**Methods:**

This retrospective study analysed the biometric measurements of 218 foetuses between 18 − 37 gestational weeks with normal MRI findings from July 2014 to August 2019, as well as 18 foetuses with VSD. The measurements included fronto-occipital diameter (FOD), biparietal diameter (BPD), and transverse cerebellar diameter (TCD). All the prenatal MRI examinations have been taken on the same 1.5 T MR unit with a standard protocol of the foetal brain. All the linear measurements of the foetal brain were obtained on the T2-weighted imaging. The distribution of measurements in 18 foetuses with VSD was plotted on centile curves.

**Results:**

The reference data were presented in mean, standard deviation, 95% predicted confidence intervals, and the 3rd, 10th, 25th, 50th, 75th, 90th, 97th centiles at each gestational age. The value of TCD in 56% (10/18 cases) foetuses with VSD was lower than the 3rd centile, and the rate for FOD and BPD was 33% (6/18 cases) and 22% (4/18 cases) separately. On the curves, most VSD cases with measurements lower than the 3rd centile were in relatively early gestational stage (≤28 weeks).

**Conclusions:**

We have presented reference linear biometry of the foetal brain by prenatal MRI from 18 to 37 gestational weeks, which could help us to interpret and monitor the brain development for foetuses with VSD and other congenital heart diseases.

**Key messages:**
1. We have presented reference linear biometry of the foetal brain by prenatal MRI from 18 to 37 gestational weeks in multiple statistical methods: mean and standard deviation; 95% predicted confidence intervals and the 3rd, 10th, 25th, 50th, 75th, 90th, 97th centiles.
2. Our data showed that the involvement of the brain in VSD may be not globally, but regionally, and the cerebellum may be more possible to be involved.
3. We speculated that the earlier the VSD diagnosed the worse the brain involved, which might suggest a poor outcome and necessary follow-up.

## Introduction

The development of the foetal head has been monitoring by ultrasound all through the gestational period. The ultrasound parameters for foetal head included biparietal diameter (BPD), transverse cerebellar diameter (TCD), and head circumference (HC). There have been already standard reference data according to gestational weeks for prenatal ultrasound screening [1]. However, all the linear measurements of the prenatal ultrasound can only reflect the size of the skull, but not the brain itself. Although brain growth is the primary driver for skull growth in the foetus and neonate, the development of the skull and brain could not be synchronous, as they both are influenced by many different factors separately. For instance, in the condition of foetal hydrocephalus, the linear measurements of the unfused skull bone could increase, while the actual brain size may decrease because of the extrinsic pressure. As the intrinsic disadvantage of the ultrasound, it is impossible to delineate the accurate borderline of the brain, because of the shadow from the skull, so a supplementary method is needed for accurate brain development monitoring.

Furthermore, monitoring for foetal brain development is essential in many conditions, congenital heart diseases, for instance. Monitoring the development of the brain in the foetus with congenital heart disease was suggested in several previous studies [2,3], and MRI was one of the optimal choices. Previous studies have compared the measurements of the brain between normal foetuses and foetuses with congenital heart diseases, but the results conflicted with each other. Some studies indicated that delayed development could be observed in foetuses with congenital heart disease [4,5], while no significant difference was found in some other studies [6,7]. The prevalence of delayed brain development may vary by the types of congenital heart diseases. Also, along with the growth of the foetus, the delay of brain development may be different by gestational age. A precise monitoring method for foetal brain is essential for the foetus with congenital heart diseases.

Magnetic resonance imaging (MRI) is a necessary imaging study as a complement for prenatal ultrasound. Substantial studies have proven that MRI could provide additional information to ultrasound in prenatal diagnosis, especially in the central nervous system [8–13]. Several studies have given out the reference values from prenatal MRI, but the coverage of the gestational weeks, sample sizes or the representation of data could not comprehensively evaluate the foetal brain [14–17]. Some studies have given some detailed information with biometry presented in mean and SD [18–21], while they were still not sufficient for the monitoring of growing tendency in follow-up MRI. Considering the clinical setting, several linear measurements by foetal MRI which are available and convenient have been included in our study, and the data was presented in three ways for monitoring the growing tendency of the foetal brain by MRI.

In our research, we provided multiple linear measurements by prenatal MRI for monitoring foetal brain development between 18 and 37 gestational weeks, presented in mean and SD, 95% predicted confidence intervals, as well as centiles. The distribution of measurements in foetuses with VSD was plotted on centile curves which were displayed in 7 centiles.

## Materials and methods

### Subjects

This retrospective study reviewed all the foetal MR imaging data in Maternal and Child Health Hospital of Hubei Province from July 2014 to August 2019, and 218 examinations of singleton pregnancies between 18 − 37 gestational weeks were included in our research for reference biometry. The MRI examinations had been performed because of maternal obesity, maternal diseases, oligohydramnios, polyhydramnios, unfavourable foetal position and increased risk of foetal pathologies, such as suspicion of foetal abnormalities by ultrasound. The inclusion criteria were: 1. No history of genetic, congenital, or developmental abnormalities in the family; 2. No abnormalities on prenatal MRI; 3. No foetal genetic abnormalities found. The gestational age at which the MRI examination was performed was calculated according to the menstrual period, which was confirmed by crown-rump length during the first trimester ultrasound, and the ultrasound biometry prior to MRI was in the normal range according to gestational age. And 18 foetuses with VSD, diagnosed by prenatal ultrasound, were included in our study for displaying the distribution of brain measurements. The protocol for this retrospective study was approved by the ethics committee of Maternal and Child Health Hospital of Hubei Province (20180306) and written informed consent was not required because of the nature of retrospective study.

### MR imaging technique

All the prenatal MRI studies were performed on a 1.5 T unit (Siemens Magnetom Espree, Siemens medical system, Germany) with a phased-array surface coil placed over maternal abdomen either in the supine or lateral position under free-breathing, except for T1-weighted imaging. No sedation or contrast agent was used on either mother or foetus. All the MRI was performed within 48 h after ultrasound examination.

All the measurements were obtained on T2-weighted half-Fourier acquisition single-shot turbo spin echo sequences in axial, coronal and sagittal planes (repetition time: 1200 ms; echo time: 143 ms; slice thickness: 4 mm; field-of-view: 260 × 260mm; matrix: 256 × 256; acquisition time: 19 s).Axial fast low-angle shot (FLASH) T1-weighted gradient echo (GRE) breath-hold sequences (TR: 2000 milliseconds; TE: 3.19 milliseconds; slice thickness: 5.5 mm; flip angle: 15°; FOV: 250 × 250 mm; matrix: 256 × 256; TA: 26 s) and axial diffusion-weighted imaging (DWI) sequence (TR: 4000 milliseconds; TE: 114 milliseconds; slice thickness: 3 mm; FOV: 400 × 400 mm; matrix: 192 × 192; TA: 28 s; b-factor: 0 and 700 s/mm2) were obtained. The duration of the MRI examination was less than 30 min.

### Image processing and analysis

**Figure 1.**
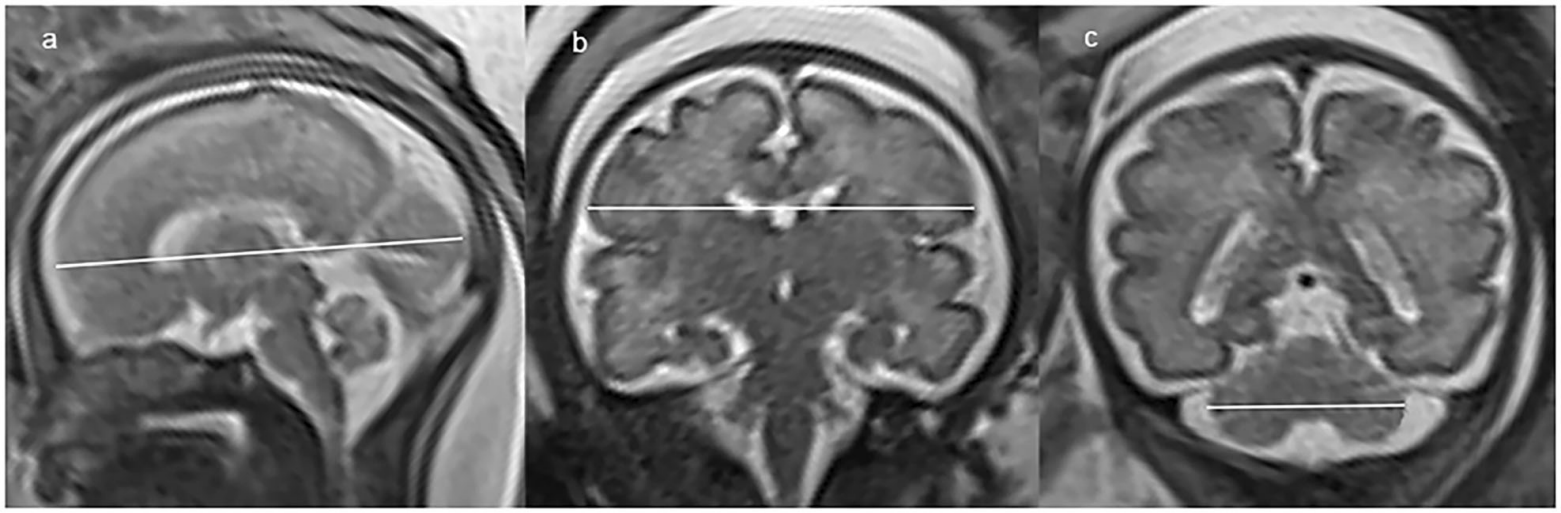
Sagittal and coronal T2-weighted imaging showing the measurements of FOD (a), BPD (b) and TCD (c) of brain itself in our study.

**Figure 2.**
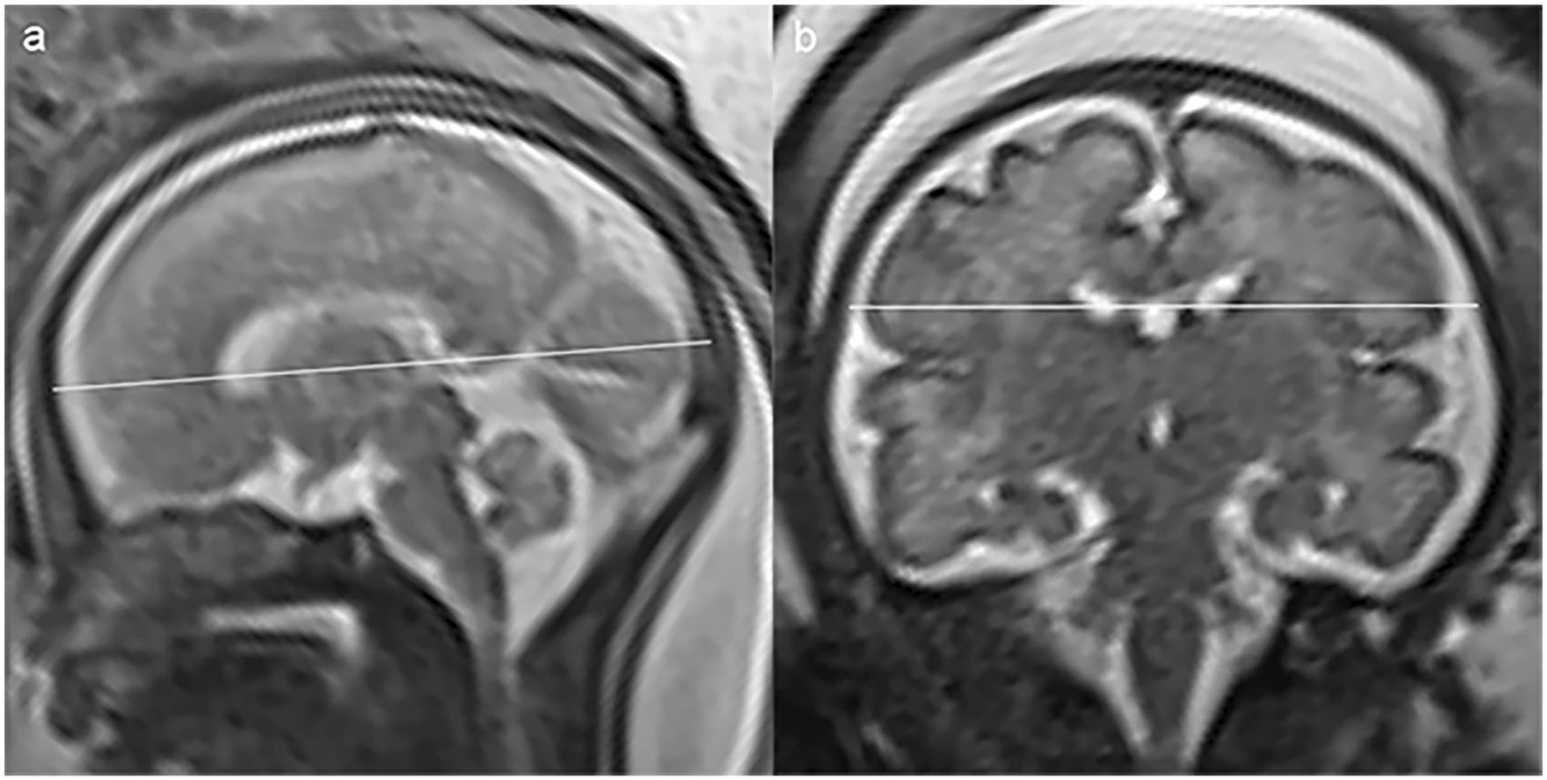
Sagittal and coronal T2-weighted imaging showing the measurements of bFOD (a) and bBPD (b) in our study.

Image analysis was performed on PACS workstation by an advanced paediatric radiologist with 23 years’ experience who was aware of the patient information. All examinations were evaluated for biometry measurement with respect to gestational week. The brain biometric parameters that were measured in our study included: cerebral fronto-occipital diameter (FOD), measured on the mid-sagittal slice as the distance between the extreme points of the frontal and occipital lobes (Figure 1(a)); cerebral biparietal diameter (BPD), measured on coronal slice at the level of the temporal horns of the lateral ventricles as the greatest transverse diameter of the cerebrum (Figure 1(b)); transverse cerebellar diameter (TCD), measured on coronal slice at the level of the atria as the greatest transverse diameter of the cerebellum (Figure 1(c)). The skull biometric parameters were measured as well: bone fronto-occipital diameter (bFOD), measured on the same slice of FOD as the distance between the two internal tables of skull (Figure 2(a)); bone biparietal diameter (bBPD), measured on the same slice of BPD as the distance between the two internal tables of skull (Figure 2(b)). Each measurement was taken twice on the T2-HASTE sequence, and the average of each measure was regarded as the best measure and used for statistical analysis.

### Statistical analysis

SPSS software (version 19), LMSChartmaker Pro software (version 2.54), and LMSgrowth (Microsoft Excel add-in written) were used for statistical analysis in our study. Worm plots and Q tests within the LMSChartmaker were used to carry out the normal distribution test for each measurement. Spearman’s rank correlation coefficient was applied to assess the correlation between measurements. A confidence level of 0.05 was considered to be significant. The mean values and SDs were calculated for the FOD, BPD, and TCD at each gestational week for all the subjects. Regression analysis was used to represent the mean and predicted 95% confidence intervals for FOD, BPD, and TCD against gestational weeks. The highest adjusted R2 value and residuals analysis were used to decide the best-fit model. Also, we compared our measurements of FOD, BPD, and TCD with published MRI data by correlation coefficient. The differences in mean values between previous MRI data and our data were displayed in a scatter diagram (data in the previous study minus data in our study).

LMSChartmaker Pro software (version 2.54) and LMSgrowth (Microsoft Excel add-in written) together were used to calculate values of the 3rd, 10th, 25th, 50th, 75th, 90th and 97th centiles at each gestational week according to our measurements in smooth curves based on the LMS method. And the brain measurements in foetuses with VSD were plotted on centile curves.

## Results

### Patient characteristics

**Figure 3.**
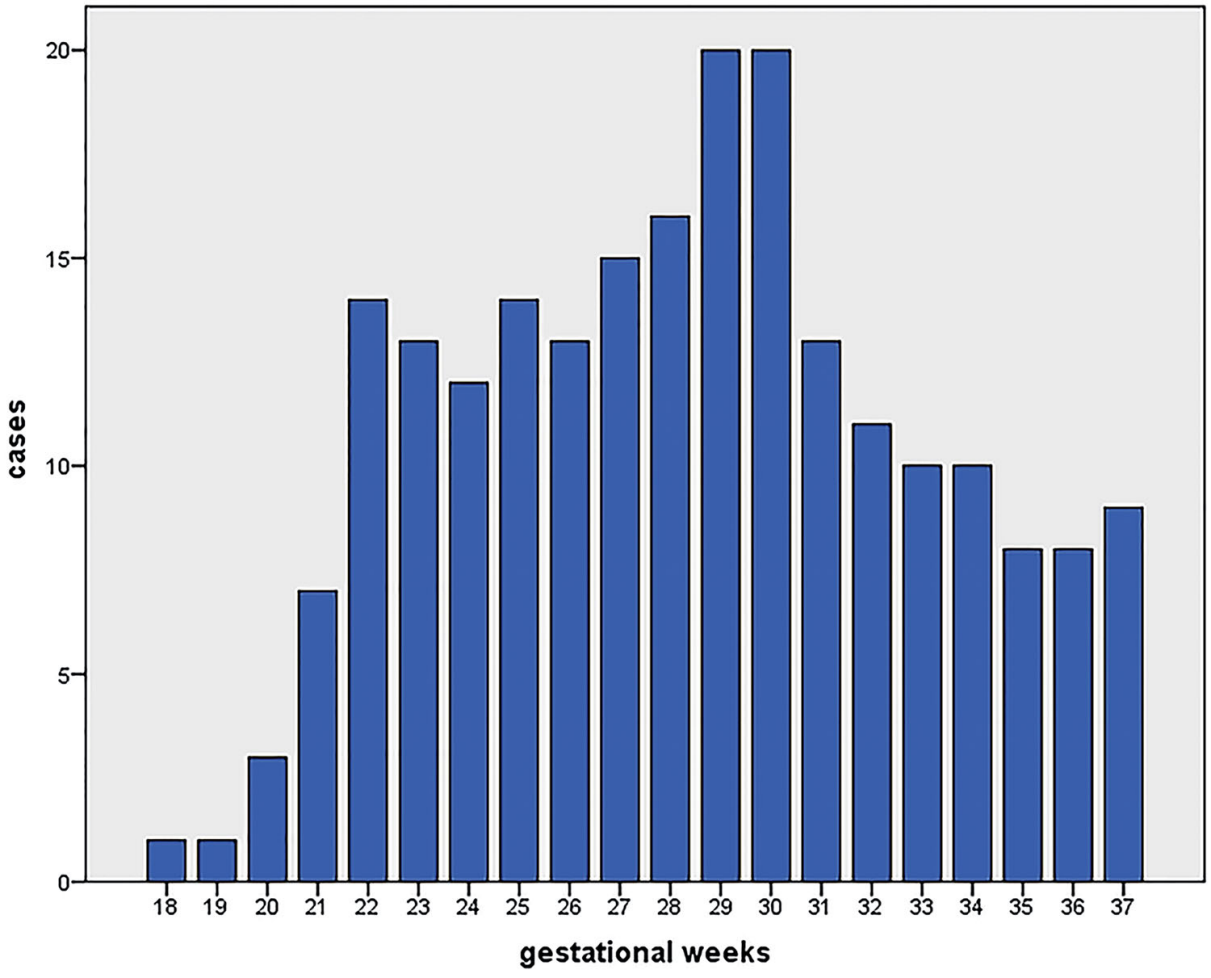
Distribution of 218 subjects included in our study by gestational age at the time of prenatal MRI examination.

**Figure 4.**
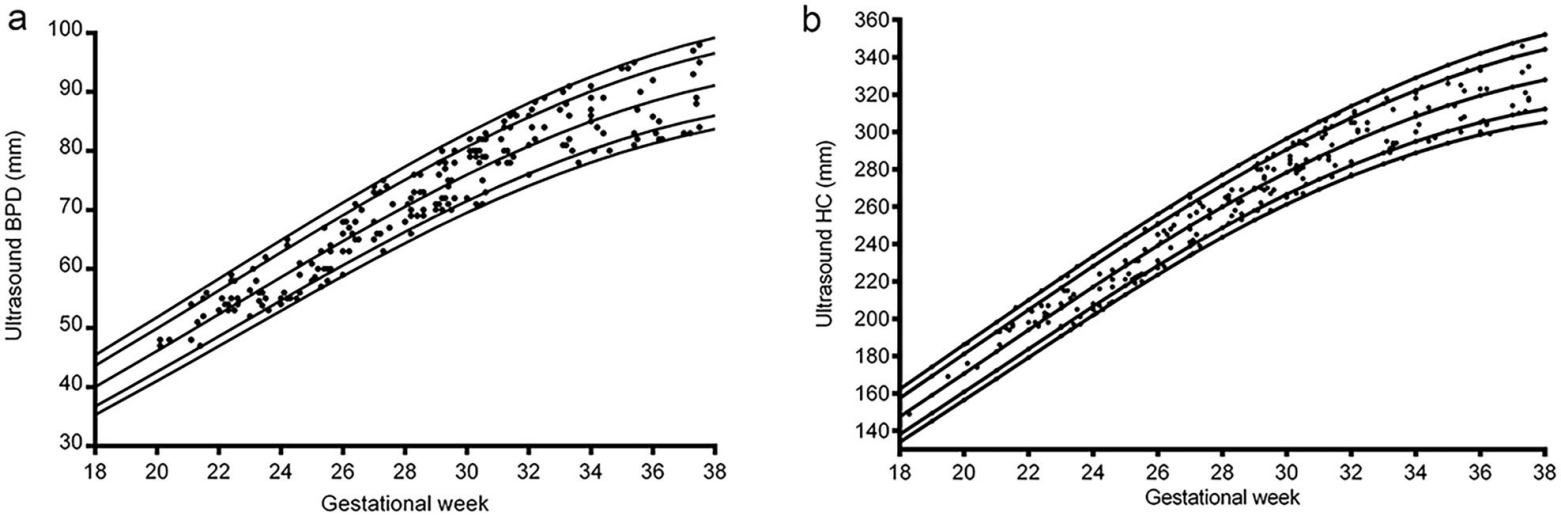
BPD and head circumference (HC) from ultrasounds on foetal growth curves for Asian.

**Figure 5.**
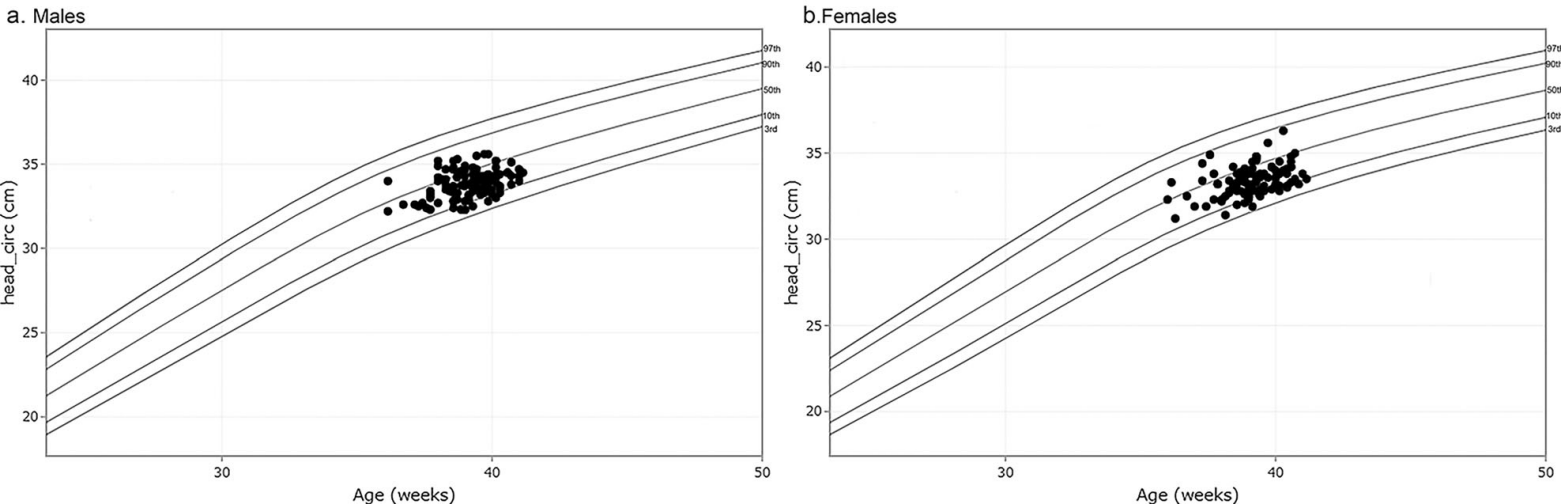
The head circumference at birth against gestational weeks on Fenton growth curves.

There were 218 foetuses included in our study for reference biometry. The numbers of foetuses at each gestational age are shown in Figure 3. The mean age of pregnant women was 29.1 years (arrange from 18 to 44 years), and the mean gestational age of foetuses was 28 + 6weeks (arrange from 18 + 3weeks to 37 + 5 weeks) at the time of the MRI examination. The foetal measurements, BPD and HC from ultrasounds, were presented in Figure 4 on foetal growth curves for Asian recommended by the National Institute of Child Health and Human Development (NICHD) [22]. The head circumference at birth against gestational weeks on Fenton growth curves was presented in Figure 5, for males and females separately [23].

There were 18 foetuses with VSD were included for displaying the distribution of their brain measurements. They were sent for MRI at the time they were first diagnosed as VSD by prenatal ultrasound. The mean age of pregnant women was 28.9 years (arrange from 20 to 44 years), and the mean gestational age of foetuses was 27 + 2weeks (arrange from 22 + 3weeks to 36 weeks).

### The mean, SD, and predicted 95% confidence intervals of FOD, BPD and TCD

**Figure 6.**
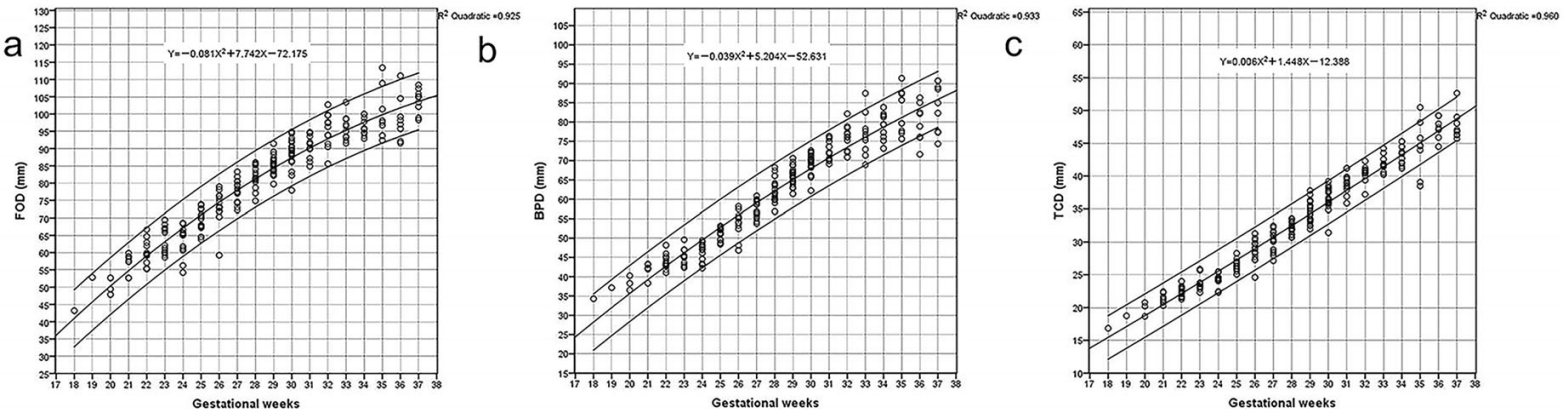
The mean and predicted 95% confidence intervals for FOD (a), BPD (b) and TCD (c).

The mean and SD of FOD, BPD, and TCD at each gestational age were displayed in Table 1, except for the 18 and 19 gestational weeks because of its limited cases. The mean and predicted 95% confidence interval of FOD, BPD, and TCD were shown in Figure 6, as well as the regression equations. Based on the highest adjusted R2 value and residuals analysis, the quadratic regression model was chosen to represent the relationship between measurement and gestational age.

**Table 1.** The mean, SD, minimum and maximum of FOD (a), BPD (b) and TCD(c).

GW	Cases	Mean	SD	Min	Max
**a. FOD**					
18	1	43.2	NULL	NULL	NULL
19	1	52.8	NULL	NULL	NULL
20	3	50.0	2.5	47.9	52.7
21	7	57.6	2.3	52.6	59.8
22	14	59.4	3.6	55.2	66.7
23	13	63.5	3.8	58.6	69.4
24	12	63.3	4.6	54.2	68.6
25	14	69.3	3.2	63.8	73.9
26	13	73.2	4.9	59.2	78.9
27	15	77.6	3.3	72.3	83.3
28	16	81.8	3.2	74.9	86.0
29	20	85.0	2.7	79.7	91.4
30	20	88.7	4.1	77.9	94.9
31	13	90.4	3.0	84.9	94.8
32	11	95.3	4.9	85.6	102.7
33	10	96.0	3.6	91.6	103.4
34	10	96.4	2.6	92.9	100.0
35	8	100.3	7.4	92.4	113.4
36	8	98.7	6.5	91.6	111.1
37	9	103.8	3.5	98.3	108.4
**b. BPD**					
18	1	34.3	NULL	NULL	NULL
19	1	37.1	NULL	NULL	NULL
20	3	38.3	1.9	36.5	40.3
21	7	41.9	1.7	38.3	43.3
22	14	43.8	1.8	41.1	48.2
23	13	45.1	2.0	42.4	49.6
24	12	46.3	2.4	42.2	49.4
25	14	50.9	1.6	48.4	53.2
26	13	53.9	3.3	46.8	58.1
27	15	57.4	2.2	53.8	60.9
28	16	62.2	3.1	56.9	68.2
29	20	66.1	2.4	61.4	70.6
30	20	69.5	2.5	62.2	72.6
31	13	72.7	2.5	69.1	76.4
32	11	76.0	3.3	70.9	82.1
33	10	76.7	5.4	68.9	87.5
34	10	78.9	3.4	73.2	83.8
35	8	82.8	5.9	75.6	91.4
36	8	79.8	5.0	71.6	86.3
37	9	83.9	6.3	74.4	90.7
**c. TCD**					
18	1	16.8	NULL	NULL	NULL
19	1	18.8	NULL	NULL	NULL
20	3	19.9	1.1	18.7	20.8
21	7	21.4	0.8	20.3	22.4
22	14	22.5	0.8	21.3	24.0
23	13	23.8	1.2	22.3	25.9
24	12	24.2	1.0	22.3	25.5
25	14	26.5	0.8	25.1	28.3
26	13	28.7	1.8	24.6	31.3
27	15	30.2	1.7	27.1	32.5
28	16	32.2	1.0	30.7	33.6
29	20	34.5	1.7	32.0	37.8
30	20	36.6	1.7	31.4	39.2
31	13	38.9	1.5	35.9	41.2
32	11	40.4	1.3	37.2	42.3
33	10	41.9	1.2	40.2	44.2
34	10	43.0	1.3	41.2	45.3
35	8	44.4	4.1	38.5	50.5
36	8	47.3	1.6	44.5	49.2
37	9	47.6	2.1	45.7	52.6

GW, gestational weeks; SD, standard deviation; Max, maximum; Min, minimum.

### The comparison between our study and previous data

**Figure 7.**
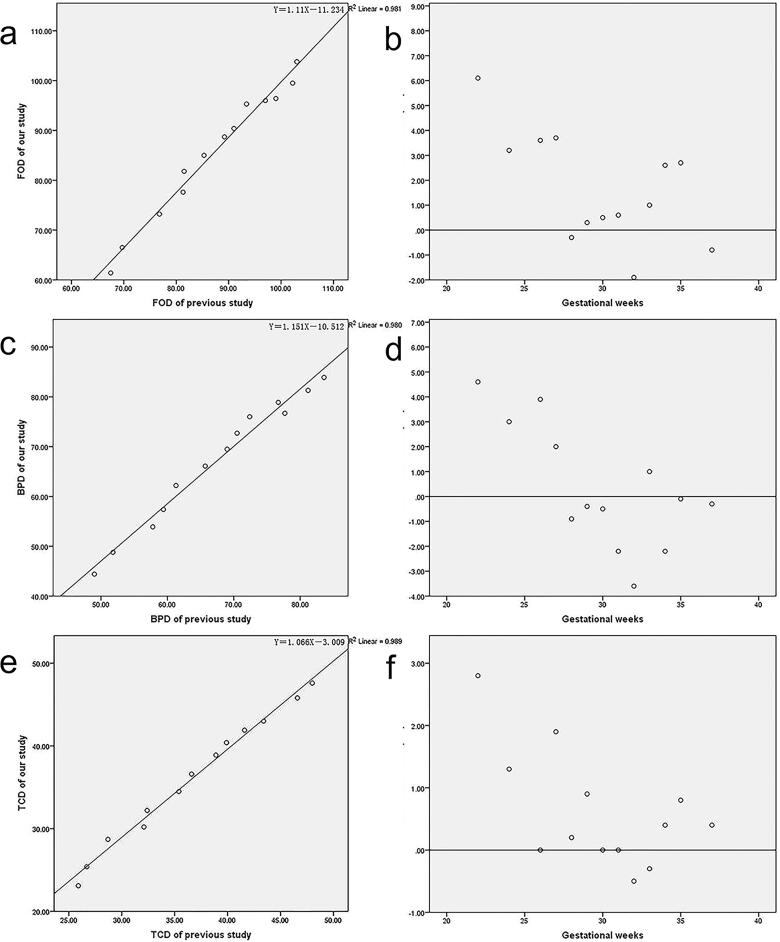
The correlation and differences in mean values of FOD (a, b), BPD (c, d) and TCD (e, f) between our study and published MRI data [13] from 22 to 37 gestational weeks.

According to the groups of gestational age which the previous study had provided [24], the comparisons of FOD, BPD, and TCD between our study and published MRI data from 22 to 37 gestational weeks were displayed in Figure 7. The correlation between our study and the previous study performed well. The value of BPD was higher in our study than the previous study in the late gestational period (Figure 7(d)).

### The centiles of FOD, BPD, and TCD

**Figure 8.**
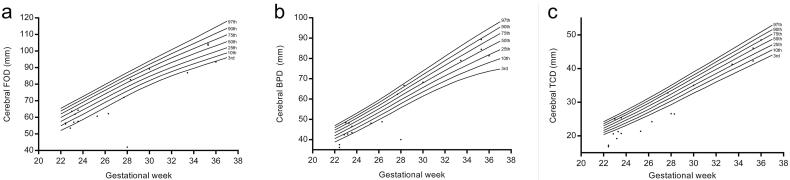
The distribution of brain measurements in foetuses with VSD on centile curves.

The 3rd, 10th, 25th, 50th, 75th, 90th, 97th centiles of FOD, BPD, and TCD calculated by LMS methods for foetuses from 22 to 37 gestational weeks were shown in Table 2, as there were not adequate cases for 18 − 21 gestational weeks. The distribution of brain measurements in the foetuses with VSD was plotted on centile curves (Figure 8).

**Table 2.** The 3rd, 10th, 25th, 50th, 75th, 90th, 97th centiles of FOD (a), BPD (b) and TCD (c).

Gestational weeks	3rd	10th	25th	50th	75th	90th	97th
**a. Cerebral Fronto-Occipital Diameter (mm)**							
22	52.1	54.9	57.5	59.9	62.0	63.8	65.4
23	55.3	58.4	61.0	63.4	65.6	67.4	69.0
24	58.7	61.8	64.5	67.0	69.2	71.0	72.6
25	62.3	65.4	68.0	70.6	72.8	74.6	76.2
26	65.9	69.0	71.6	74.1	76.3	78.1	79.8
27	69.6	72.5	75.1	77.6	79.8	81.7	83.4
28	73.1	75.9	78.4	81.0	83.3	85.2	87.0
29	76.5	79.2	81.7	84.2	86.6	88.6	90.5
30	79.5	82.2	84.7	87.3	89.8	92.0	94.0
31	82.4	85.0	87.5	90.3	92.9	95.2	97.4
32	85.0	87.6	90.2	93.1	95.9	98.4	100.8
33	87.4	90.0	92.7	95.7	98.7	101.5	104.2
34	89.6	92.3	95.1	98.3	101.5	104.5	107.5
35	91.8	94.5	97.4	100.8	104.3	107.6	111.0
36	93.9	96.7	99.7	103.2	107.0	110.7	114.5
37	96.0	98.9	102.0	105.7	109.8	113.7	118.0
**b. Cerebral Biparietal Diameter (mm)**							
22	38.8	40.5	41.9	43.4	44.7	45.9	46.9
23	41.4	43.1	44.7	46.2	47.6	48.8	49.9
24	44.1	45.8	47.5	49.1	50.6	51.8	53.0
25	46.9	48.7	50.4	52.1	53.7	55.0	56.2
26	49.8	51.6	53.4	55.2	56.8	58.2	59.5
27	52.7	54.6	56.5	58.3	60.1	61.6	63.0
28	55.7	57.7	59.6	61.6	63.4	65.1	66.6
29	58.6	60.7	62.6	64.8	66.8	68.5	70.2
30	61.4	63.5	65.7	67.9	70.1	72.0	73.8
31	64.0	66.3	68.6	71.0	73.4	75.4	77.4
32	66.4	68.9	71.4	74.0	76.6	78.9	81.0
33	68.7	71.4	74.1	77.0	79.8	82.2	84.6
34	70.6	73.7	76.7	79.9	82.9	85.6	88.1
35	72.3	75.9	79.3	82.8	86.1	88.9	91.5
36	73.7	78.0	81.8	85.7	89.2	92.1	94.8
37	74.8	79.9	84.3	88.5	92.3	95.4	98.1
**c. Transverse Cerebellar Diameter (mm)**							
22	20.4	21.0	21.6	22.3	23.0	23.6	24.2
23	21.7	22.4	23.1	23.9	24.6	25.3	25.9
24	23.1	23.9	24.7	25.5	26.3	26.9	27.6
25	24.6	25.4	26.3	27.1	28.0	28.7	29.4
26	26.1	27.0	27.9	28.9	29.7	30.5	31.2
27	27.7	28.7	29.7	30.6	31.6	32.3	33.1
28	29.3	30.4	31.4	32.4	33.4	34.2	35.0
29	31.0	32.1	33.2	34.3	35.3	36.2	37.0
30	32.7	33.9	35.0	36.1	37.2	38.1	39.0
31	34.3	35.6	36.8	38.0	39.1	40.0	40.9
32	36.0	37.3	38.5	39.8	41.0	42.0	42.9
33	37.6	39.0	40.3	41.6	42.8	43.9	44.9
34	39.2	40.6	42.0	43.4	44.7	45.8	46.9
35	40.8	42.3	43.7	45.2	46.6	47.8	48.9
36	42.4	43.9	45.4	47.0	48.4	49.7	50.9
37	44.0	45.6	47.1	48.7	50.3	51.6	52.9

### The correlation between different measurements

**Figure 9.**
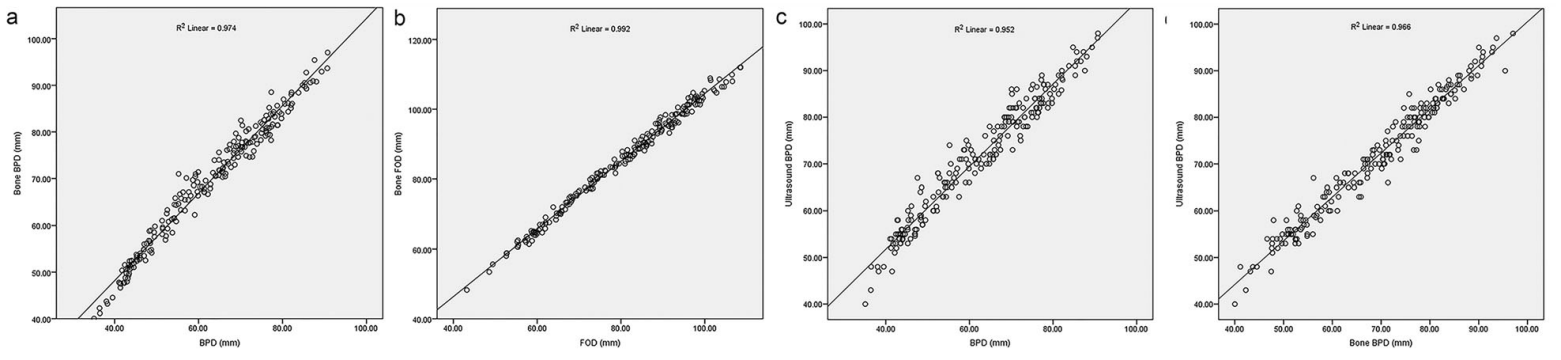
The correlation between BPD and bBPD (a), FOD and bFOD (b), BPD and ultrasound BPD, bBPD and ultrasound BPD in our study.

The scatterplots were produced plotting cerebral MRI measurements against skull MRI measurements alongside the data obtained from 218 foetuses, as well as MRI measurements against ultrasound BPD (Figure 9 for details). The correlation coefficient was 0.995 between FOD and bFOD, and 0.988 between BPD and bBPD, and 0.974 between BPD and ultrasound BPD, and 0.982 between bBPD and ultrasound BPD, with all *p* < .05.

## Discussion

Our data presented a prenatal MRI biometry of the foetal brain from a large cohort (*n* = 218) including FOD, BPD, and TCD from 18 to 37 gestational weeks. In order to represent our data from multiple perspectives, we showed mean, SD, maximum, minimum, 95% predicted confidence intervals (along with the regression equation) and the 3rd, 10th, 25th, 50th, 75th, 90th, 97th centiles of our measurements. The data of our study reflected the development of the brain itself, but not the skull. As we know, ultrasound biometry could only reflect the size of the skull, while MRI provides the possibility to display the actual size of the brain. It is important to know the brain size when we have to evaluate the development of the brain itself, for instance, in the condition of congenital heart diseases. The gestational age arranged from 18 to 37 weeks, as it’s recommended that prenatal MRI should be performed after 18 gestational weeks, not only for safety, but also for valuable additional information it could provide after 18 gestational weeks [25].

So far, not much study of prenatal brain MRI measurements with large cohort represented by multiple statistical methods has been reported. Some of them were based on the measurement of the skull in comparison with ultrasound, not for the brain itself [26]. Some of them were based on volume measurement which was time-consuming [26,27]. Furthermore, most of the studies had limited ranges of gestational ages and small sample sizes [14,15,28–30]. In our clinical practice, FOD, BPD and TCD are the most frequently used values; also, they are adequate and efficient to evaluate the size of the cerebrum and the cerebellum in the setting of pervasive application. During the interpretation of the prenatal MRI biometry, reference data were essential for evaluating the development tendency of the foetal brain, while the mean and SD were insufficient in monitoring the brain development. As biometric measurement is the basic evaluation for foetal brain imaging diagnosis and the first step to exclude abnormalities, we sought to provide a reference data set in multiple statistical methods for dynamically monitoring foetal brain development.

In order to prove the credibility and validity of our data, we presented the ultrasound biometry on foetal growth curves for Asian, which was recommended by the National Institute of Child Health and Human Development (NICHD) [22], as well as head circumference at birth against gestational weeks on Fenton growth curves [23]. And we compared the correlation between cerebral measurements and bone measurements in our study, as well as MRI measurements and ultrasound measurements, which represented a significant correlation. The correlation between cerebral measurements and bone measurements by MRI and ultrasound could not only validate the accuracy of our data, but also the integrity between the parenchymal structures and skull. Furthermore, we compared part of our data with Garel’s study [24] from 22 to 37 gestational weeks. The correlation between our study and Garel’s data performed well. The mean values of FOD, BPD and TCD were smaller in our study than the relative values in Garel’s study during the second trimester, especially for FOD and TCD with maximum differences around 6.1 mm for FOD, 4.6 mm for BPD, and 2.8 mm for TCD separately. While the value of BPD was higher in our study than in Garel’s during the third trimester. It may be due to the sample from a different population and the insufficient sample size, which suggested that reference data for different population was necessary by foetal MRI.

One advantage of our study was a sample size of 218 foetuses from 18 to 37 gestational weeks with at least seven subjects for almost all gestational ages. It allowed the calculation for centiles from 22 to 37 gestational weeks. No centiles were obtained for 18 − 21 gestational weeks because of limited cases (1, 1, 3, and 7 separately). Additionally, our data could provide reference data for the foetal brain in multiple perspectives, such as mean and SD, 95% predicted confidence intervals, as well as centiles for the evaluating of the foetal brain development.

In our study, the distribution of brain measurements by MRI in foetuses with VSD was displayed on centile curves, which could be interpreted intuitively. The value of TCD in 56% (10/18 cases) foetuses with VSD was lower than the 3rd centile, and the rates for FOD and BPD were 33% (6/18 cases) and 22% (4/18 cases) separately. Rollins has suggested that the involvement of brain size in the foetus with hypoplastic left heart syndrome or transposition of the great arteries was in a region-specific pattern [31]. Regional involvement of the brain was also observed in foetuses with single ventricle congenital heart disease [32]. Our data showed that the involvement of the brain in VSD was also not globally, but regionally, and the cerebellum may be more possible to be involved. Smaller cerebellar volume was observed in the foetuses with congenital heart disease in Olshaker’s study [33]. It may suggest that the involvement of the brain could not only be attributed to the blood supply, the genetic or environmental factors may also contribute [34]. On the centile curves, most VSD cases with measurements lower than the 3rd centile were in relatively early gestational stage (≤28 weeks). We speculated that the earlier the VSD diagnosed the worse the brain involved, which might suggest a poor outcome and necessary follow-up.

We had several limitations. First, our data were obtained retrospectively, obstetric information on the maternal status was not obtained, and some of the foetuses were suspected for abnormalities by ultrasound. So it may not properly represent the normal population. Also, we did not have the follow-up of the foetuses included in our study, such as postnatal imaging or evaluation of cognitive development. However, previous study has proven that the diagnostic accuracy was high for prenatal MRI [35]. Third, volume measurements could reflect the development more accurately, which would be included in our future research. Next, we are going to collect the outcome of the foetuses in order to verify cognitive development. This will help us to improve our reference data and understand the differences between normal foetuses and the foetuses with VSD.

In conclusion, we have presented reference linear biometry of the foetal brain by prenatal MRI from 18 to 37 gestational weeks by multiple statistical methods, which will help us to interpret and monitor the foetal brain development better, for foetuses with congenital heart diseases, for instance.

## Data Availability

All datasets generated for this study are included in the article.
